# Saliva Proteomics Analysis Offers Insights on Type 1 Diabetes Pathology in a Pediatric Population

**DOI:** 10.3389/fphys.2018.00444

**Published:** 2018-04-26

**Authors:** Eftychia Pappa, Heleni Vastardis, George Mermelekas, Andriani Gerasimidi-Vazeou, Jerome Zoidakis, Konstantinos Vougas

**Affiliations:** ^1^Department of Operative Dentistry, School of Dentistry, National and Kapodistrian University of Athens, Athens, Greece; ^2^Department of Orthodontics, School of Dentistry, National and Kapodistrian University of Athens, Athens, Greece; ^3^Proteomics Laboratory, Foundation of Biomedical Research of the Academy of Athens, Athens, Greece; ^4^Diabetic Centre of P&A Kuriakou Children's Hospital, Athens, Greece

**Keywords:** type 1 diabetes, salivary proteome, glycemic regulation, children, mass spectrometry

## Abstract

The composition of the salivary proteome is affected by pathological conditions. We analyzed by high resolution mass spectrometry approaches saliva samples collected from children and adolescents with type 1 diabetes and healthy controls. The list of more than 2000 high confidence protein identifications constitutes a comprehensive characterization of the salivary proteome. Patients with good glycemic regulation and healthy individuals have comparable proteomic profiles. In contrast, a significant number of differentially expressed proteins were identified in the saliva of patients with poor glycemic regulation compared to patients with good glycemic control and healthy children. These proteins are involved in biological processes relevant to diabetic pathology such as endothelial damage and inflammation. Moreover, a putative preventive therapeutic approach was identified based on bioinformatic analysis of the deregulated salivary proteins. Thus, thorough characterization of saliva proteins in diabetic pediatric patients established a connection between molecular changes and disease pathology. This proteomic and bioinformatic approach highlights the potential of salivary diagnostics in diabetes pathology and opens the way for preventive treatment of the disease.

## Introduction

Salivary analysis is increasingly used as a clinical tool in dentistry, internal medicine, endocrinology, pediatrics, immunology, and psychology. It allows the reliable assessment of a clinically relevant number of drugs, hormones, and antibodies (Lima et al., 2010; Wu et al., 2010; Yeh et al., 2010; Lima-Aragão et al., 2016). Saliva offers an attractive alternative to blood samples, particularly in children and the elderly, where blood sample collection often reduces compliance to follow-up (Lima et al., 2010; Yeh et al., 2010). Moreover, salivary diagnostics may provide a cost-effective tool in monitoring oral and systemic health and disease in large populations, especially when repeated sampling is necessary (Samaranayake, 2007; Lima et al., 2010; Yeh et al., 2010).

Whole saliva contains specific proteins produced by the salivary glands whereas crevicular fluid, mucosal tissue, bacteria, viruses, and fungi also contribute to the composition of the saliva proteome (Dodds et al., 2005). The acinar cells of the salivary glands are responsible for the secretion of more than 85% of salivary proteins, and the glandular duct cells secrete proteins with vital biological functions such as growth factors and immunoglobulins (Vitorino et al., 2004). Approximately 40–50% of the salivary proteome consists of small proteins and peptides (Amado et al., 2010) derived by proteolysis in the oral cavity (Vitorino et al., 2010). Several studies indicate the effect of systemic diseases on salivary variables and outline their importance in understanding the pathogenesis of the disease (Tishler et al., 1997; Cho et al., 2010; Javaid et al., 2016; López-Pintor et al., 2016).

It has been previously shown that salivary proteomes present alterations in type 1 and type 2 diabetic patients. Rao et al. characterized the salivary proteome in subjects with pre-diabetes, type 2 diabetes and healthy controls, identifying a total of 487 unique proteins, of which 65 were found to be differentially expressed in saliva from patients with type 2 diabetes vs. controls (Rao et al., 2009). Border et al showed that the differences in saliva proteomes were observed in edentulous patients with type 2 diabetes as well, where 96 peptides corresponding to 52 proteins were found to be differentially expressed between diabetic and non-diabetic controls (Border et al., 2012). Moreover, salivary peptidomic modifications were identified in patients with type 1 diabetes, when compared to healthy controls, indicating down-regulation of peptides involved in oral cavity host defense in these patients (Cabras et al., 2010). Proteomic changes associated with hyperglycemia were determined by a label-free proteomic approach, demonstrating that there is a correlation between specific proteins and HbA1c levels in patients with diabetes (Bencharit et al., 2013).

Chronic hyperglycemia is the critical factor for the development and progression of diabetic complications. Cardiovascular disease (CVD), chronic inflammation, nephropathy, retinopathy, and peripheral neuropathy are the most common complications of the disease. The risk of death from CVD in adults with poorly-controlled type 1 diabetes is 10 times greater than in the general population (Katz et al., 2015). While optimal glycemic control is crucial for the reduction of CVD risk, adolescents and young adults demonstrate higher Hb1Ac levels compared to other age groups, thus they are at high risk for early complications (Katz et al., 2015). The molecular mechanisms influencing the severity of diabetic complications are not fully understood in the early stages of the disease. Proteomic profiling of clinical samples is a powerful tool for the identification of altered biochemical pathways and bio markers of disease states (Donaghue et al., 2009; Katz et al., 2015).

In this study we investigated the proteomic profile of whole saliva by high resolution mass spectrometry in type 1 diabetic children and adolescents with satisfactory and poor glycemic control in comparison with sex- and age-matched healthy controls The aim of the present work was to characterize the salivary proteome of type 1 diabetes patients in order to identify differentially expressed proteins compared to control subjects, infer deregulated biological pathways, and evaluate the relevance of the findings in the context of diabetes pathophysiology.

## Materials and methods

### Study design and clinical data

The study protocol and written consent forms were approved by the Medical Ethics Committee of the Faculty of Medicine of the University of Athens (according to the instructions of the Declaration of Helsinki) and all experimental methods were performed in accordance with the relevant guidelines and regulations. The study protocol was explained to both parents and children, and informed written consent to participate in the study was obtained from a parent. Diabetic patients were enrolled at the Diabetic Centre of P&A Kuriakou, Athens, Children's Hospital and controls at the respective Pediatric Department. Subjects were all examined by a group of internal medicine physicians during their regular follow-up.

During examination, the assessment of complications consisted of clinical assessment by the endocrinologist, neurologist, and ophthalmologist accordingly. Screening for retinopathy, microalbuminuria, and neuropathy took place during the examination. The presence/diagnosis of any diabetic complication was considered as an exclusion criterium for the participation in this study. Percentage of hemoglobin Hb1Ac was determined with the use of the HPLC (HA8140) Instrument. BMI, blood pressure and cholesterol values were additionally measured by routine clinical laboratory methods and details on all clinical parameters are shown in Table 1.

**Table 1:** Subjects' demographics (*p-value < 0.05).

	**G1**	**G2**	**Ctrl**
Age(yrs), mean (SD)	14.5 ± 1.7	14.1 ± 1.3	14.9 ± 1.8
Gender, n (M/F)	5/7	5/7	5/7
Time with DM1 (yrs),	5.8 ± 1.9	6.4 ± 2.8	-
HbA1c % (mmol/mol)	9.7 ± 0.7*(83)	6.2 ± 0.4*(44)	4.2 ± 0.4*(22)
BMI (kg/m^2^)	22.9 ± 4	20.7 ± 5	24.3 ± 3
Blood Pressure (mmHg)	82 ± 5	79 ± 4	85 ± 5
Diastolic Blood Pressure (mmHg)	67 ± 3	63 ± 3	70 ± 4
Systolic Blood Pressure (mmHg)	113 ± 4	109 ± 3	114 ± 3
Total cholesterol (mg/dL)	165 ± 10	160 ± 12	168 ± 15
LDL cholesterol (mg/dL)	92 ± 6	88 ± 5	94 ± 8

*Mean and standard deviation values are reported*.

A total of 36 subjects participated in the study and were allocated in three groups. Group 1 (G1) consisted of 12 type 1 diabetic patients with poor glycemic control, group 2 (G2) of 12 patients with satisfactory glycemic control while the control group (Ctrl) comprised of 12 healthy subjects sex-and-aged-matched accordingly. HbA1c values ≥7.5% (58 mmol/mol) indicated poor glycemic control for type 1 diabetes.

### Standardized sample collection

The composition of saliva varies considerably depending on different conditions (Castagnola et al., 2012). In order to effectively control potential sources of variability, the following protocol was applied:

Prior to the day of the examination, participants were advised not to eat or drink 1 h before their scheduled appointment. All saliva samples were collected between 10:00 a.m. and 12:00 p.m., to minimize any inter-individual variation of saliva composition associated with circadian rhythms. Unstimulated whole saliva was collected from all participants. In case the subject became stressed or began to cry, the sample was discarded. Gingival index was recorded and subjects with oral inflammation were excluded from the study. During saliva collection, a specialized dentist examined all participants using as exclusion criteria the presence of gingivitis or any clinical signs of oral infammation. The gingival index (Löe, 1967) was recorded during a clinical examination; a score below 1 was a prerequisite for the subjects of all three groups (Löe, 1967). Whole saliva was collected from the anterior floor of the oral cavity using a soft plastic aspirator for less than 1 min and transferred to a plastic tube. Collection tubes were stored on ice at all times during the examination, and 3.6% v/v protease inhibitors (Roche) were used in order to prevent proteolytic degradation of salivary proteins.

### Protein digestion and iTRAQ labeling

The protein present in each saliva sample was precipitated through acetone precipitation, hence by mixing four volumes of ice-cold acetone with one volume of saliva sample, overnight incubation at −20°C and consequent centrifugation for 20 min at 4,000 g at 4°C. The precipitated protein was re-dissolved in 200 μL dissolution buffer 0.5 M triethylammonium bicarbonate (TEAB) with extensive vortex mixing and pulsed probe sonication for 20 sec. Undissolved material was separated from the protein solution with centrifugation at 13,000 rpm for 10 min. The total of 36 samples were separated in six batches, each batch containing six samples two from each group (G1, G2, & Ctrl). Each batch was processed separately as described in the following section and the samples of each batch were iTRAQ-labeled as described in Supplementary Table 1.

For each sample a total protein amount of 50 μg was measured with Bradford assay (Bio-Rad Protein Assay) according to manufacturer's instructions and was diluted with the addition of dissolution buffer up to a final volume 20 μL. Cysteine disulfide bonds were reduced with the addition of 2 μL reducing reagent 50 mM tris-2-carboxymethyl phosphine (TCEP) followed by 1 h incubation in heating block at 60°C. Cysteine residues were blocked by the addition of 1 μL 200 mM methanethiosulfonate (MMTS) in isopropanol and 10 min incubation at room temperature. Samples were diluted with 14 μL ultrapure water and 6 μL of proteomics grade trypsin (Roche Diagnostics) solution 500 ng/μL were added for overnight digestion at 37°C. A 50 μL volume of isopropanol was added to each iTRAQ-8plex reagent vial and after vortex mixing the content of each iTRAQ vial was transferred to each sample tube. Labeling reaction was completed in 2 h at room temperature, samples were pooled and the whole mixture was dried with a speedvac concentrator. The labeled peptide samples were stored at −20°C until they were analyzed by high-pH Reversed Phase (RP) Chromatography.

### High-pH reverse phase (RP) peptide fractionation

High-pH RP C18 fractionation of the iTRAQ-8plex labeled peptides was performed on the Dionex P680 pump equipped with PDA-100 photodiode array detector using the Waters, XBridge C18 columm (150 × 4.6 mm, 3.5 μm). Mobile phase (A) was composed of 0.05% v/v ammonium hydroxide aqueous solution and mobile phase (B) was composed of 100% v/v acetonitrile, 0.05% v/v ammonium hydroxide. The peptide pellet of each batch was dissolved in 200 μL mobile phase (A) with bath sonication. Sample was centrifuged at 13,000 rpm for 5 min and the supernatant solution was injected through a 200 μL sample loop. The separation method was as follows: for 15 min isocratic 5% (B), for 10 min gradient up to 35% (B), for 5 min gradient up to 80% (B), for 5 min isocratic 80% (B) at a flow rate 0.4 mL/min. Signal was monitored at 280, 254, and 215 nm and the column temperature was set to 30°C. Eight fractions were collected and were finally dried with speedvac concentrator for 4–5 h and stored at −20°C until the LC-MS analysis.

### LC-MS analysis

All LC-MS experiments were performed on the Dionex Ultimate 3000 UHPLC system coupled with the high resolution nano-ESI Orbitrap-Elite mass spectrometer (Thermo Scientific). Individual high-pH RP peptide fractions were reconstituted in 30 μL loading solution composed of 2% acetonitrile, 0.1% formic acid in ultra pure water. A 5 μL volume was injected and loaded for 8 min on the Acclaim PepMap 100, 100 μm × 2 cm C18, 5 μm, 100 Å trapping column with the ulPickUp Injection mode with the loading pump operating at flow rate 5 μL/min. The peptides were eluted under a 315 min gradient from 2% (B) to 33%(B). Flow rate was 300 nL/min and column temperature was set at 35°C. Gaseous phase transition of the separated peptides was achieved with positive ion electrospray ionization applying a voltage of 2.5 kV. For every MS survey scan, the top 10 most abundant multiply charged precursor ions between m/z ratio 300 and 2,200 and intensity threshold 500 counts were selected with FT mass resolution of 60,000 and subjected to HCD fragmentation. Tandem mass spectra were acquired with FT resolution of 15,000. Normalized collision energy was set to 33 and already targeted precursors were dynamically excluded for further isolation and activation for 45 s with 5 ppm mass tolerance.

### Database search

The collected HCD tandem mass spectra were submitted to the cited Tandem search engine (Craig and Beavis, 2004) implemented on the Trans Proteomic Pipeline (TPP) software version 4.6 for peptide and protein identifications (Deutsch et al., 2015). All spectra were searched against a UniProt Fasta file containing 20,200 human reviewed entries. The TPP included the following parameters: Precursor Mass Tolerance 10 ppm, Fragment Mass Tolerance 0.05 Da, Oxidation of M (+15.995 Da) was the only Dynamic Modification considered and Static Modifications were iTRAQ8plex at any N-Terminus, K, Y (+304.205 Da) and Methylthio at C (+45.988 Da). The Peptide and Protein Prophet TPP-modules were used for the determination of the confidence level for peptide and protein identifications with decoy database searching controlling the False Discovery Rate (FDR) at 1 and 5% at the peptide and protein levels respectively. The Libra module of the Trans Proteomic Pipeline (Pedrioli, 2010) module was utilized for peptide and protein quantification through the iTRAQ reporter ions. The signal intensity of the individual reporter ions was normalized in LIBRA and further normalization to account for unequal loading was performed in R(R Core Team, 2016). For each iTRAQ batch, each individual iTRAQ ion reporter was normalized according to the following formula:

Normalized iTRAQ reporter ion intensity (i) = iTRAQ reporter ion intensity (i)/Sum of all 6 iTRAQ reporter ion intensities.

Based on the assumptions that (a) all samples in each batch were equally loaded and (b) the majority of proteins are not differentially expressed across comparisons, we infer that the average normalized iTRAQ reporter intensity for all identified proteins per sample should equal 16.67% (100/6). If the average of normalized values deviates significantly from the aforementioned value, this is due to unequal loading. All iTRAQ reporter ions in each batch were corrected for an equal loading by setting the average of each iTRAQ reporter ion distribution at 16.67%.

Our analysis was based on high confidence protein identifications (peptide level FDR<1%, protein level FDR<5%, as controlled by TPP) (Kinsinger et al., 2012).

### iTRAQ reporter ion intensities meta-analysis

The normalized iTRAQ reporter ion intensities of all 36 samples were used for the identification of differentially expressed proteins across all the possible comparisons among the three groups, namely G1 vs. Ctrl, G2 vs. Ctrl, and G1 vs. G2. The normalized intensities of each protein which were found to be normally distributed according to the Kolmogorov-Smirnov test for normality were tested for differential expression using the *t*-test. Following that the average expression ratios of each protein for all the aforementioned comparisons were calculated and consequently log-2 transformed and centered. A second differential protein expression calculation was performed based solely on the magnitude of change indicated by the log2ratios, where *p*-values indicating differential expression were then calculated. For both tests *p* < 0.05 was considered significant. The above procedures were carried out in the R language(R Core Team, 2016).

### Pathway analysis

The significantly deregulated proteins from the previous procedure were imported into QIAGEN's Ingenuity® Pathway Analysis and were analyzed for biological context against the IPA Knowledge base (IPA®, QIAGEN Redwood City, www.qiagen.com/ingenuity). In IPA analysis differentially expressed proteins were considered those with log2ratio *p*-value < 0.05. The pathway enrichment analysis was performed by using as reference database not the complete human proteome but only the identified proteins in our analysis. Further biological insight along with suggestions for potential clinical interventions was obtained from the L1000CDS^2^ database (Vempati et al., 2014).

### Sample preparation for MRM

Multiple reaction monitoring (MRM) was used for quantitation of analytes. 12 different samples were pooled to final 100 μg total protein saliva extract for each group (G1,G2,Ctrl) and each pooled sample was analyzed in three technical replicates. Each pooled sample was diluted to a final volume 100 μL with urea buffer (8M urea, 50 mM NH_4_HCO_3_) followed by reduction (10 mM DTE) and alkylation (40 mM Iodoacetamide). The samples were then diluted in to final volume of 2 mL with 50 mM NH_4_HCO_3_ in order to dilute urea to final concentration below 1 M. Trypsin was added to enzyme: protein ratio 1:100 and incubated overnight. After trypsinization, the samples are desalted by zip-tip. Finally, the desalted samples were dried (speedVac) and reconstituted in appropriate volume of mobile phase A (water, 0.1% formic acid) and heavy peptide mix (Final concentration approximately 10 ng/μl of each peptide) to final protein concentration of 1 μg/μL and measured by LC/MRM as described below.

### MRM assay design and method development

#### Proteotypic peptide selection

The human spectral library was searched using the software Skyline and the peptide atlas repository to identify proteotypic peptides for the 24 proteins selected for validation (Deutsch, 2010). Spectral information of proteotypic peptides for the design of MRM experiments exist for all proteins and 1–3 peptides per protein were selected (Supplementary Table 2). The final transition selection (Supplementary Table 3) was based on the quality of the MS/MS spectrum of each peptide in the human spectral library, downloaded from NIST (National Institute of Standards and Technology, http://www.nist.gov/), and on the score and number of observations in MS-based proteomics experiments as provided from PeptideAtlas.

#### LC-MRM set-up

Liquid chromatography was performed using an Agilent 1200 series nano-pump system (Agilent Technologies, Inc., Palo Alto, CA), coupled with a C18 nano-column (150 mm × 75 μm, particle size 3.5 μm) from AB Sciex. Peptide separation and elution was achieved with a 40 min 5–35% ACN/water 0.1% FA gradient at a flow rate of 300 nL/min. Four microliters of each sample were injected. Each pooled sample was analyzed in triplicates.

Tryptic peptides were analyzed on an AB/MDS Sciex 4000 QTRAP with a nano- electrospray ionization source controlled by Analyst 1.5 software (Sciex). The mass spectrometer was operated in MRM mode, with the first (Q1) and third quadrupole (Q3) at 0.7 unit mass resolution. Three to five transitions were recorded for the endogenous (light) and heavy peptides. In total 501 transitions for 44 peptides were monitored in two methods. Optimum collision energies for each transition were automatically calculated by the Skyline software.

#### Data analysis and quantification

Isotope labeled peptides (^13^C and ^15^N) identical to the endogenous ones were used in order to determine the specificity of the detection. Data analysis was performed using Skyline software and all chromatograms were manually inspected to ensure high data quality and accurate peak picking. More specifically, two criteria were used to determine high confidence peptide identification: the correlation with the spectral library and the co-elution of isotope labeled and endogenous peptides (Feng and Picotti, 2016). Finally, the sum of peak areas of at least three transitions per endogenous peptide was used for quantification (Supplementary Table 3).

## Results

### Salivary proteome

Saliva samples from 36 children and adolescents aged 6–18 were divided in three groups: Group 1 (G1) consisted of 12 with well-regulated type 1 diabetes, group 2 (G2) of 12 with poorly-regulated type 1 diabetes and 12 healthy subjects were the control group (Ctrl). The level of blood glycated hemoglobin (HbA1c) was accessed and presented normal values in controls (below 5.9%) and ranged from 6 to 12% in type 1 diabetic patients. HbA1c values ≥7.5% indicated poor metabolic control for type 1 diabetes while values<7.5% were considered as well-control of the disease (Table 1). BMI, blood pressure, cholesterol values were additionally measured and details on all clinical parameters are shown in Table 1.

**Figure 1:**
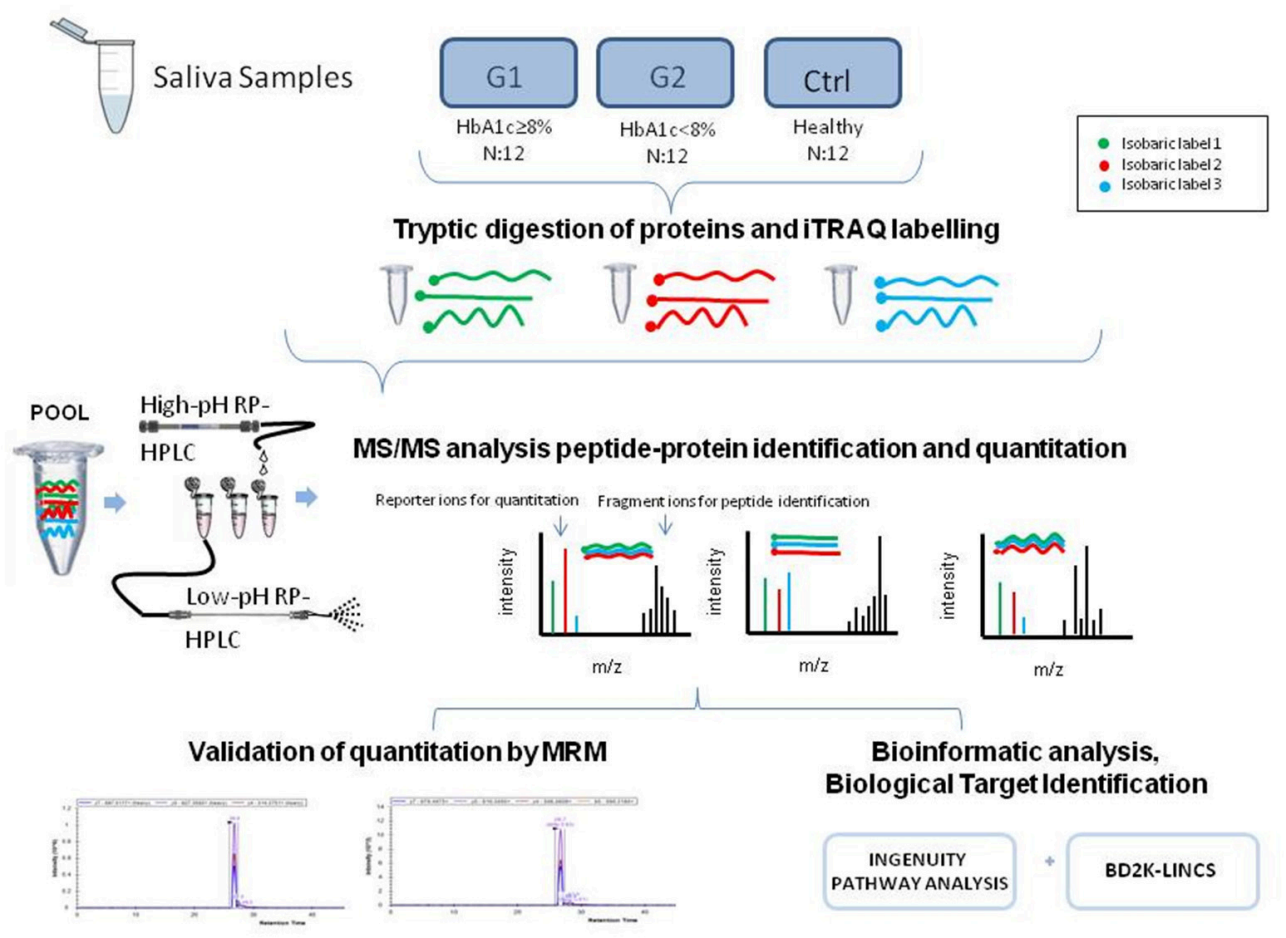
The graphical abstract outlines the proteomic and bioinformatic analysis of saliva samples.

The experimental outline for proteomic and bioinformatic analysis of the saliva samples is illustrated in Figure 1.

The proteomic analysis yielded 22028 peptides that were confidently identified (FDR < 1%) (Supplementary Table 4) and quantified by the iTRAQ reporter ions. These peptides corresponded to 4876 individual confident protein identifications (FDR< 5%) (Kinsinger et al., 2012) (Supplementary Table 5).

**Figure 2:**
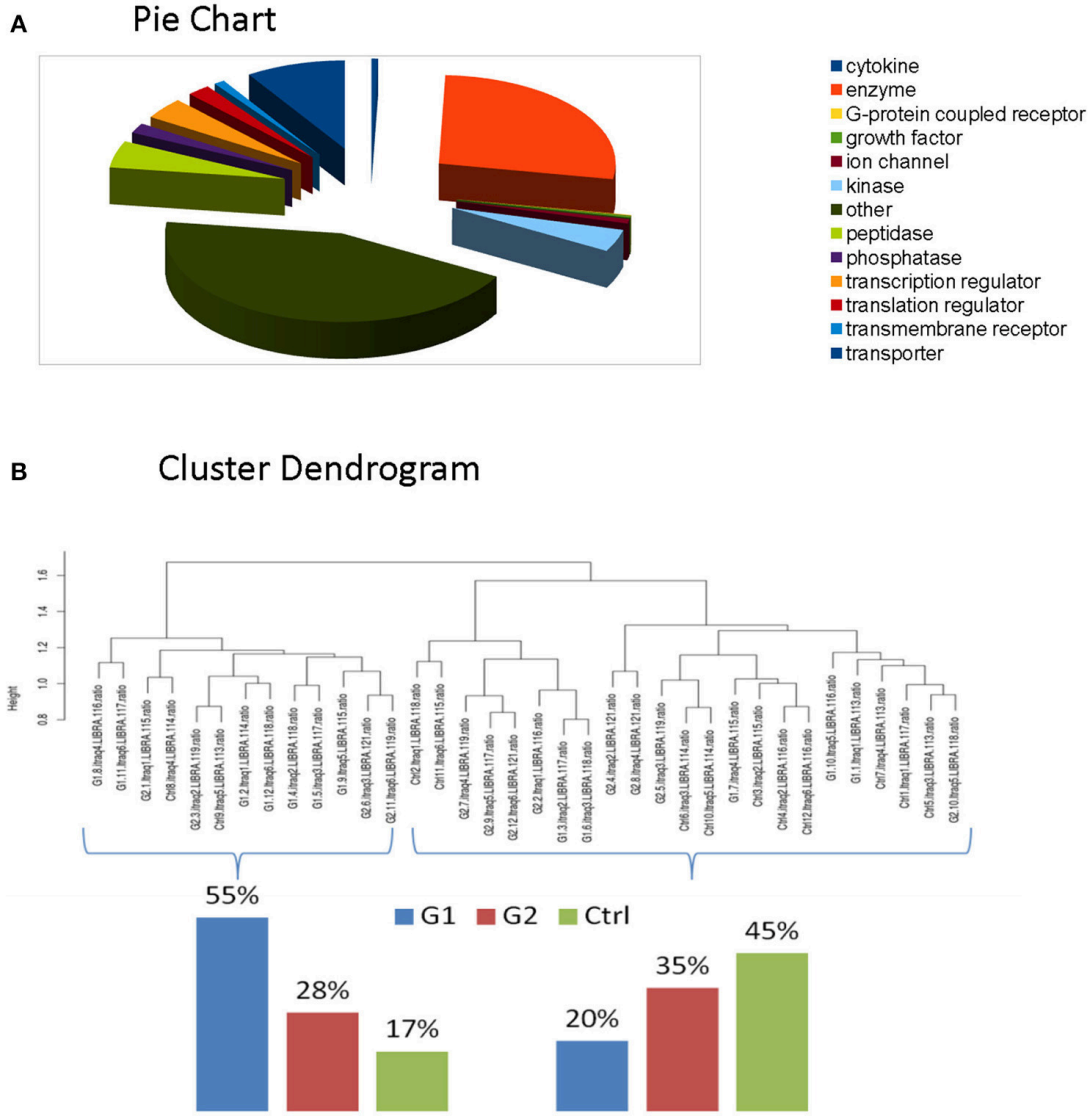
**(A)** Functional classification of these proteins revealed that Enzymes and Cytokines were the main functional groups of the salivary proteome. **(B)** Clustering indicated that total proteomic profile is capable of distinguishing poorly controlled subjects from well-controlled and healthy subjects. The latter ones present similarities, as expected.

For the comparative analysis among groups (poorly-regulated type 1 diabetic patients, well-regulated type 1 diabetic patients & healthy controls), only the proteins being present at a percentage equal or greater than 70% of the samples (9≥12) in each group were selected. The total protein number considered for analysis was reduced to 2031 proteins (Supplementary Table 6). Functional classification of these proteins revealed that Enzymes and Cytokines were the main functional groups of the salivary proteome. (Figure 2A, Supplementary Table 7-Functional IPA).

Protein identifications for each individual iTRAQ batch and the protein prophet analysis through which the false discovery rate was controlled are presented in Supplementary Table 10. Furthermore, Supplementary Table 6 presents the 2031 proteins for analysis and the protein identification probabilities for each protein across all six iTRAQ batches. Notably, each protein identification across all iTRAQ batches is confirmed by at least one protein identification probability with FDR<1% as calculated from the data presented in Supplementary Table 6.

In our experimental design, each batch consisted of 6 reporter ions because we intended to maintain an identical cross batch experimental design (Supplementary Table 1); each iTRAQ batch consisted of 2 G1, 2 G2, and 2 Ctrl with iTRAQ reporter ions randomly assigned to eliminate possible reporter ion intensity bias. The identical cross batch design in combination with Libra normalization eliminates the need for an internal standard in each batch, simulating cross group comparison in label free quantitation experiments. Each sample has been analyzed individually on the mass spectrometer and the precursor ion intensities have been normalized against the total ion current. The advantage of our iTRAQ based cross batch comparison is that our analyzed samples present lower interexperimental variability due to the iTRAQ multiplexing.

### Differential expression

We performed clustering and correlation analysis in order to determine whether quantitation from the total proteome profiling of our samples could produce a meaningful class discrimination (see the relevant Methods section). Hierarchical clustering analysis based on the Euclidean distance of the normalized iTRAQ reporter ions of the total cohort confirms that our experimental design in combination with the applied normalization and processing totally eliminated any batch effect since no batch oriented grouping was detected in any of these analyses. Particularly, clustering indicated that total proteomic profile is capable of distinguishing poorly controlled subjects from well-controlled and healthy subjects. The latter ones present similarities, as expected. (Figure 2B).

**Figure 3:**
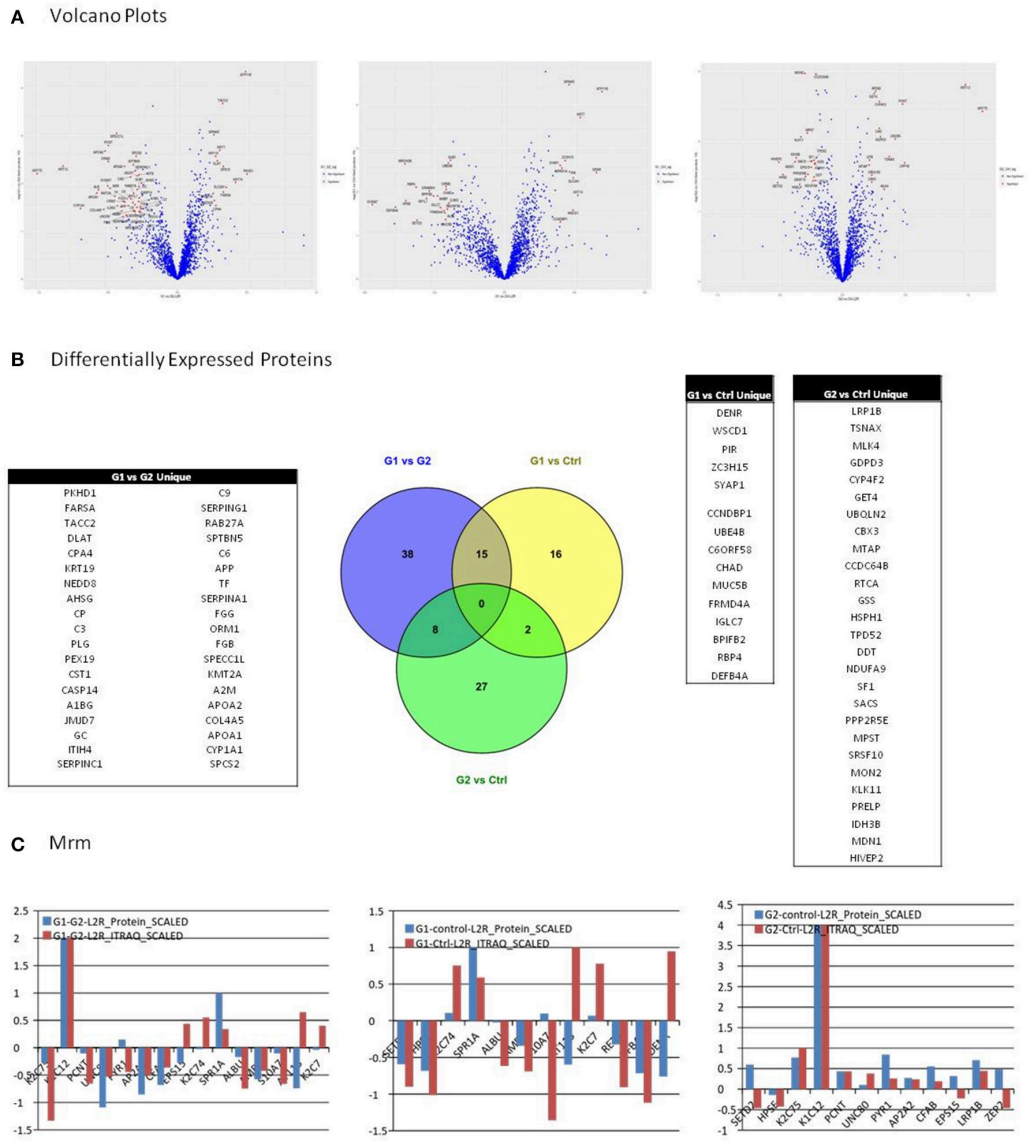
**(A)** Among possible comparisons, G1 vs. Ctrl and G1 vs. G2 yield proteins with high fold change and low *p*-values. Volcano plots present that the variance in G2 vs. Ctrl is smaller than in the other two comparisons, indicating higher similarity between G2 and Ctrl subjects. **(B)** All possible comparisons were performed among the three groups: (G1-Ctrl, G2-Ctrl, G1-G2). Thirty three proteins were found to be differentially expressed between G1-Ctrl, 37 between G2-Ctrl and 61 between G1-G2. **(C)** Multiple Reaction Monitoring (MRM) was utilized in order to validate the relative quantitation obtained by the iTRAQ technology. For each group comparison, we selected the most relevant proteins, based on differential expression and clinical relevance. The proteins selected presented low *p*-value, high fold-change, and were the most relevant to clinical pathways. In G1 vs. G2, 9 out of 12 proteins presented positive correlation between the iTRAQ and MRM quantitation. Nine out of 12 proteins presented positive correlation in iTRAQ and MRM quantitation in comparison G1 vs. Ctrl as well, whereas in G2 vs. Ctrl, 10 out of 15 presented positive correlation.

Selection of differentially expressed proteins for each pair-wise comparison, was performed by applying a double significance criterion. The first one was the *t*-test between two groups for each individual protein intensity values from iTRAQ reporter ions. The second one was the log2ratio *p*-value which corresponded solely to the magnitude of change for each protein between two groups (see the relevant Methods section). All possible comparisons were performed among the 3 groups: (G1-Ctrl, G2-Ctrl, G1-G2). Thirty-three proteins were found to be differentially expressed between G1-Ctrl, 37 between G2-Ctrl and 61 between G1-G2 (Figure 3B, Supplementary Table 8).

The following results were obtained from the proteomic analysis, for differentially expressed proteins (Table 2).

**Table 2:** The most prominent protein findings among the three comparisons.

**Protein**	**ttest_pvalue**	**l2r**	**Ratio**	**Fold change**	**Biological function**
**G1-CTRL**					
S100A7	0.019	−0.755	0.592	−1.688	Immune response
DEFB4A	0.022	−0.622	0.649	−1.539	Inflammation
A2M	0.042	−0.268	0.830	−1.204	Acute phase response, coagulation
SERPINA1	0.024	−0.287	0.819	−1.220	Atherosclerosis
LPO	0.037	−0.291	0.816	−1.224	Phagosome maturation
S100A10	0.030	0.226	1.170	1.170	Dissolution of fibrin clot
CASP4	0.015	0.255	1.193	1.193	Cell apoptosis, nephropathy
**G1-G2**					
S100A7	0.011	−0.501	0.706	−1.416	Immune response
A2M	0.012	−0.486	0.713	−1.401	Acute phase response, coagulation
C3	0.030	−0.265	0.831	−1.202	Complement
SERPING1	0.036	−0.309	0.807	−1.238	Complement
APOA1	0.015	−0.580	0.668	−1.495	LXR/FXR, atherosclerosis
SERPINA1	0.045	−0.367	0.775	−1.289	Atherosclerosis, coagulation
PLG	0.033	−0.265	0.831	−1.202	Coagulation
**G2-CTRL**					
SETD2	0.038	−0.516	0.698	−1.430	Enzyme
HIVEP2	0.019	−0.507	0.703	−1.421	Transcription regulator
HPSE	0.029	−0.474	0.719	−1.389	Enzyme
LRP1B	0.020	0.493	1.408	1.408	Transmembrane receptor
KRT75	0.003	1.113	2.163	2.163	Other

*P-value, log2ratio, and fold-change are presented, along with the pathways in which these proteins are involved. Biologically relevant findings with high statistical significance were not identified in G2 vs. Ctrl*.

G1-Ctrl down: The protein found to be most downregulated was Protein S100-A7 (fold change = −1.69) followed by Beta-defensin 4A (fold change = −1.54) and Maestro heat-like repeat-containing protein family member 2B (fold change = −1.48). HPSE, AMBP, ALB, A2M, APOA2, LPO, SERPIN, S100A2 were also found to be significantly downregulated in this comparison. G1-Ctrl up: Probable phospholipid-transporting ATPase IF (fold change = 1.47) was found to be most upregulated, followed by DENR (fold change = 1.44) and KRT7 (fold change = 1.35). SPRR1A, CASP4, S100A10, PSMB7 were also significantly upregulated in this comparison.

G2-Ctrl top3 down: The protein found to be most downregulated was SETD2 (fold change = −1.43) followed by HIVEP2 (fold change = −1.42), and HPSE (fold change = −0.56). G2-Ctrl top3 up: KRT75 (fold change = 2.76) was found to be most upregulated, followed by KRT12 (fold change = 1.97) and LRP1B (fold change = 1.41).

G1-G2 top3 down: The protein found to be most downregulated was KRT75 (fold change = −2.0) followed by KRT12 (fold change = −1.78) and CYP1A1 (fold change = −1.62). APOA1, ALB, APOA2, S100A7, A2M, AMBP, SERPING1, APOB, C3, ITIH4, CST1, CFB, AHSG were also found to be significantly downregulated in this comparison group. G1-G2 top3 up: PKHD1 (fold change = 1.42) was found to be most upregulated, followed by ATP11B (fold change = 1.41) and KRT74 (fold change = 1.34). S100A10 was also significantly upregulated, as observed in G1-Ctrl comparison.

Among possible comparisons, G1vs. Ctrl and G1 vs. G2 yield proteins with high fold change and low *p*-values. As shown in the Volcano plots (Figure 3A), the variance in G2 vs. Ctrl is smaller than in the other two comparisons, indicating higher similarity between G2 and Ctrl subjects.

### MRM validation

We utilized Multiple Reaction Monitoring (MRM) (see Methods) in order to validate the relative quantitation obtained by the iTRAQ technology. For each group comparison, we selected the most relevant proteins, based on differential expression and clinical relevance. The proteins selected presented low *p*-value, high fold-change and were the most relevant to clinical pathways. In G1 vs. G2, 9 out of 12 proteins presented positive correlation between the iTRAQ and MRM quantitation. 9 out of 12 proteins presented positive correlation in iTRAQ and MRM quantitation in comparison G1 vs. Ctrl as well, whereas in G2 vs. Ctrl, 10 out of 15 presented positive correlation (Figure 3C).

### Bioinformatic analysis

#### IPA

For biological knowledge extraction we utilized the QIAGEN's Ingenuity® Pathway Analysis Platform (see Methods). The results are shown in Tables 3, 4.

**Table 3:** Deregulated pathways identified in G1 vs. G2 comparison.

**Pathways**	***p*-value**	**Molecules**	
		**Downregulated**	**Upregulated**
Acute Phase Response Signaling	3.16^*^10^−20^	SERPING1,C3, APOA2,C9, AHSG,AMBP,CP,FGG,PLG,IL36G,ALB,APOA1, ORM1,TF, IL1RN,ITIH4,CFB,ORM2, SERPINA1,FGB,HRG,MAP2K1,A2M	
LXR/RXR Activation	2^*^10^−15^	APOB,C3,APOA2,C9,AHSG,AMBP,A1BG,ALB,IL36G,APOA1,TF,ORM1, IL1RN,ITIH4,ORM2,SERPINA1	GC
Atherosclerosis Signaling	2.9^*^10^−7^	ALB,IL36G,APOB,APOA1,ORM1,IL1RN,APOA2,ORM2,SERPINA1	PRDX6
Coagulation System	8.5^*^10^−7^	PLG,SERPINC1,SERPINA1,FGB,A2M,FGG	
Complement System	2.5^*^10^−5^	SERPING1,C3,C9,CFB,C6	
IL-12 Signaling and Production in Macrophages	4.4^*^10^−5^	ALB,APOB,APOA1,ORM1,APOA2,ORM2,SERPINA1,MAP2K1	
IL-10 Signaling	4^*^10^−3^	IL36G	BLVRA,BLVRB, IL1RN
Toll-like Receptor Signaling	5.4^*^10^−3^	IL36G	UBB,TOLLIP,IL1RN

**Table 4:** Deregulated pathways identified in G1 vs. Ctrl comparison.

**Pathways**	***p*-value**	**Molecules**	
		**Downregulated**	**Upregulated**
Acute Phase Response Signaling	9.6^*^10^−12^	APOA2,AHSG,AMBP, ALB,IL36G,TF,IL1RN,IL36RN,ORM2,SERPINA1,MAP2K3,HRG,A2M,RBP4	MYD88, IL18, CP
LXR/RXR Activation	10^−10^	IL36G,ALB,TF,IL1RN,APOA2,IL36RN,AMBP,AHSG,ORM2,SERPINA1,GC,A1BG,RBP4	IL18
Phagosome maturation	1.3^*^10^−7^	LPO, NAPG	DYNLL1,CALR,TUBA1C,ATP6V1G1,NAPA,PRDX6,EEA1,PRDX5,PRDX1
Atherosclerosis Signaling	1.6^*^10^−6^	ALB,IL36G, IL1RN,APOA2,IL36RN,ORM2,SERPINA1,RBP4	IL18,PRDX6
Toll-like Receptor Signaling	2^*^10^−5^	UBB, IL1RN,IL36RN,MAP2K3	IL36G,IL18,MYD88
IL-10 Signaling	1.2^*^10^−4^	IL36G,IL1RN,IL36RN,MAP2K3	BLVRB,IL18

In the G1 vs. Ctrl comparison, Acute phase response signaling, Atherosclerosis signaling and LXR/RXR- FXR/RXR activation were the top canonical pathways which were found to be activated, whereas molecules related to cardiotoxicity, hepatotoxicity and nephrotoxicity were identified in this comparison. For the G1 vs. G2 comparison, similar canonical pathways were found to be deregulated. Biologically relevant findings with high statistical significance were not identified in G2 vs. Ctrl.

The ingenuity pathway analysis software is a standard software for determining deregulated pathways connected to disease (Jiménez-Marín et al., 2009). The *p*-value reported reflects the proportion of proteins deregulated in the pathway (high proportions correspond to low *p*-values), and also the proportion of proteins of a pathway in the differentially expressed protein list for each comparison (high proportion of proteins that belong to a specific pathway in the whole list of differentially expressed proteins yield low *p*-values).

#### LINCS

We introduced in L1000CDS^2^, a search engine of gene expression signatures from the LINCS L1000 dataset, the differentially expressed (up & down-regulated) proteins of Comparison G1 vs. G2. Among the agents that most efficiently reversed that phenotype, the top hit was BRD-K01868942, a serotonin receptor antagonist (Supplementary Table 9).

## Discussion

### Why saliva?

The issue of children's compliance in monitoring their serum glucose has shifted researchers' focus toward saliva, a non- invasive, easily collected biological fluid which presents an attractive alternative to blood samples(Lima et al., 2010; Yeh et al., 2010) Analysis of saliva may provide insights to biological processes for patients with diabetes and could potentially reveal early complications through biological mechanisms activated long before the appearance of clinical symptoms of the disease. It is important to note that blood collection in a pediatric population can cause poor compliance of patients, thus saliva collection for glycemic monitoring is an attractive alternative. (Kaczor-Urbanowicz et al., 2017)

### HbA1c threshold

Salivary proteomic changes of Type 1 diabetes were analyzed based on the HbA1c regulation. Elevated HbA1c predicts long-term microvascular and macrovascular complications and is the only biomarker of glycemic control with strong outcome data (Rewers et al., 2009). A target range of <7.5% (58 mmol/mol) is recommended, following the ISPAD Consensus Guidelines, for all age-groups. Of all age-groups, adolescents show the poorest performance in achieving optimal glycemic control, an observation which is in accordance with the physiological, hormonal challenges, and the increased independence in diabetes care during this period (Rewers et al., 2009; Spencer et al., 2010).

### Diabetes and gingival inflammation

A large number of studies suggest that diabetes is associated with an increased prevalence, extent and severity of oral inflammatory diseases such as gingivitis and periodontitis (Lalla et al., 2007; Giuca et al., 2015). Whole saliva is a combination of the secretions of the major and minor salivary glands, together with the gingival crevicular fluid (Giannobile et al., 2009) and is the biological fluid used in the present study for proteomic analysis. Taking into account the lack of compliance in oral hygiene during adolescence, subjects with oral inflammation were excluded from the study. For that purpose, the gingival index (Löe, 1967) was recorded by a specialized dentist during a clinical examination; a score below 1 was a prerequisite for the subjects of all three groups (Löe, 1967). Thus, the proteomic analysis of saliva in our study highlights differences due to diabetic pathology and excludes the contribution of oral inflammation.

In our study, the presence of gingival inflammation was an exclusion criterion for all the participants. However, the pathway analysis indicated a deregulation of the mechanisms involved in inflammation, immune response and IL-12 signaling in poorly-controlled diabetic adolescents. Inflammation and immune response play key role in periodontal diseases such as gingivitis and periodontitis, which are considered to be the most common complications of diabetes (Karjalainen and Knuuttila, 1996). Additionally, the osteolytic role of the proinflammatory cytokine interleukin IL-12 was recently found to be involved in the pathogenesis of periodontal diseases (Issaranggun Na Ayuthaya et al., 2018). Differentially expressed proteins in these pathways, that could contribute to periodontal disease, are presented in Table 2.

### Subjects demographics

BMI values in control group are on the threshold between normal and overweight for 15-year-old adolescents, measured at 24 kg/m^2^, a value which is not surprising taking into account the high prevalence of juvenile obesity in Greece (Krassas et al., 2001; Tzotzas et al., 2008). The aforementioned BMI differentiation in the control group did not contribute to the variance of the overall dataset at the extent of having a detectable effect.

### High confidence data

For the purposes of this study, saliva from a large cohort of 36 individuals was analyzed utilizing peptide labeling & multiplexing technology (iTRAQ) for high-confidence protein identification and quantitation. In order to exceed the iTRAQ multiplexing limitation (eight samples in a single run), we devised a controlled, we devised a controlled experimental design and data normalization scheme in order to combine six individual iTRAQ 6-plex batches (see Methods and Figure 1-Graphical Abstract). Furthermore the protein identification was performed with the Trans Proteomic Pipeline (Pedrioli, 2010) (TPP) where the False Discovery rate was controlled both at the peptide and at the protein level, selecting for analysis only the proteins present in all the samples. Finally the expression level as calculated from the iTRAQ reporter ions for a large number of proteins was validated by MRM which is a highly sensitive and specific mass spectrometry technique. In our study, MRM was performed on three pooled samples. Pooling samples could mask individual sample variability within each group. Moreover, aberrantly high or low levels of specific proteins in individual samples could influence the concentration in the entire group. However, the results obtained by the MRM approach are in general agreement with the proteomics data derived from the individual samples. These initial findings have to be further confirmed by analysis of an independent cohort of saliva samples.

Our approach did not involve multiple testing correction of *p* values, which is a limitation in our study. However, a double criterion was used for the selection of differentially expressed proteins, namely *t*-test *p*-value and log2ratio *p*-value. Proteins were considered significant when both *p*-values < 0.05. This approach is equivalent to a volcano-plot based selection of differentially expressed proteins, with the added value of being systematic and not empirical since rigid statistical criteria based on actual numerical values (both *p*-values < 0.05) where used as a selection threshold.

Despite the limitations, the process described above, produced a highly reliable data set which gave us the opportunity for in depth proteomic analysis of type 1 diabetes utilizing saliva, an easily and non-invasively acquired biological sample. Compared with previous proteomic studies, the present one provides a significantly higher number of reliable protein identifications (total 2031) (Rosa et al., 2012). Rao et al. has previously identified a total of 491 proteins in saliva of type 2 diabetic subjects (Rao et al., 2009), Cabras et al. detected 120 salivary components using HPLC-ESI-MS analysis of whole human saliva of children with type 1 diabetes (Cabras et al., 2010), while 148 proteins were detected using pooled samples per type of diabetes by Bencharit et al. (2013) The number of proteins confidently identified in the present study is comparable to the total number of 2290 proteins that Loo et al. report by combining salivary proteomic datasets from several studies (Loo et al., 2010).

### Studies on salivary proteome in diabetes

It has been previously shown that salivary proteomes present alterations in type 1 and type 2 diabetic patients. Rao et al. characterized the salivary proteome in subjects with pre-diabetes, type 2 diabetes and healthy controls. A total of 487 unique proteins was identified, of which 65 were found to be differentially expressed in saliva from patients with type 2 diabetes vs. controls (Rao et al., 2009). The majority of the differentially expressed proteins were associated with pathways regulating metabolism and immune response, similarly to the findings of our study (Rao et al., 2009). Salivary proteomes also presented differences in edentulous patients with type 2 diabetes, where 96 peptides corresponding to 52 proteins were found to be differentially expressed between diabetic and non-diabetic controls (Border et al., 2012). Moreover, salivary peptidomic modifications were identified in patients with type 1 diabetes, when compared to healthy controls, indicating down-regulation of peptides involved in oral cavity host defense in these patients (Cabras et al., 2010). Proteomic changes associated with hyperglycemia were determined by a label-free proteomic approach, showing that there is a correlation between specific proteins and HbA1c levels in patients with diabetes (Bencharit et al., 2013). In accordance with the findings of our study, this analysis demonstrated alterations in the salivary proteomic values of various serum originating proteins including albumin, complement C3 and alpha2-macroglobulin, related to increased levels of HbA1c (Bencharit et al., 2013).

### Deregulated pathways

Based on high confidence data the bioinformatics analysis yields biologically significantly deregulated pathways. The lack of significant differences, observed in G2 vs. Ctrl for pathways, is in accordance with the clinical data available for these two groups. Indeed, satisfactory glycemic control is the key factor for prevention of diabetic complications (Lebovitz et al., 2006). G2 and Ctrl subjects presented similar proteomic profiles, which is in accordance with their respective health status. The similarity of the proteomic profiles of Ctrl and G2 subjects is the main reason for which when these two “healthy” groups are compared with the deregulated patients (G1), there is considerable overlap in the two lists of differentially expressed proteins. This is illustrated in Figure 3B. However, as shown in the same figure, there are also unique differentially expressed proteins. These differences are probably due to the diverging genetic background of Ctrl subjects when compared to G2- well-regulated T1D patients.

Contrary to G2 vs. Ctrl comparison, common and biologically relevant proteins are identified in the two comparisons G1 vs. Ctrl and G1 vs. G2. Differential expression of proteins in the G1 group, led to activation of molecular pathways related to pathological complications, as shown in Tables 3, 4. Acute phase response signaling, LXR/RXR activation network, atherosclerosis and coagulation pathway, immune response, and toll-like receptor signaling appear to be deregulated in poorly controlled patients.

Regulation of the mechanisms controlling inflammation and synthesis of acute phase proteins is impaired by hyperglycaemia and the direct relationship between hyperglycaemia, inflammatory process and oxidative stress contributes to the development of diabetic complications (Gordin et al., 2008; Beisswenger, 2012). Additionally, functional defects of the immune system have been correlated with the metabolic control of diabetic patients and are related to increased susceptibility of these patients to infections (Moutschen et al., 1992). The pathway analysis indicated a deregulation of the key mechanisms involved in inflammation and immune response in poorly-controlled diabetic adolescents.

Liver X receptors (LXRs), transcription factors of a nuclear hormone receptor family, play an important role in metabolic regulation. They control cholesterol and glucose homeostasis in the body and recent studies in type 2 diabetic models have shown that LXRs regulate insulin secretion and biosynthesis via control of glucose and lipid metabolism in pancreatic b-cells (Efanov et al., 2004; Ding et al., 2014). In our study, deregulation of LXR/RXR pathway in G1 could reflect the inadequate metabolic control of the disease.

Toll-like receptors (TLRs), another signaling pathway which was found to be deregulated in poorly controlled patients in our study, are proteins that play key role in the innate immune system. These immune receptors are able to recognize microbial molecules, detect infections and initiate antimicrobial host defense responses. According to new data, autoimmune diabetes is found to be triggered by the innate immune pathways and TRLs are the mediators of this mechanism (Zipris, 2010).

**Figure 4:**
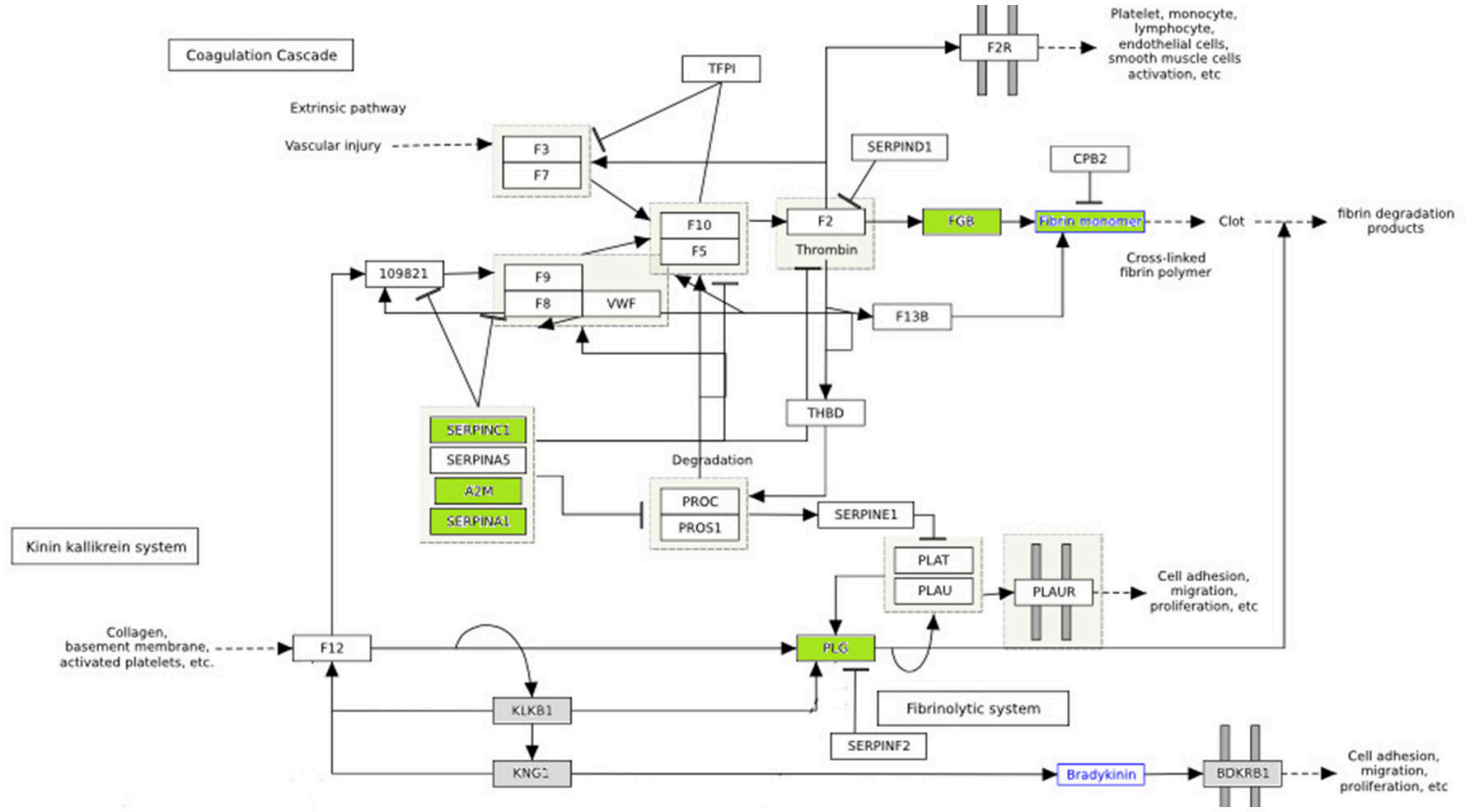
The effect of diabetes on fibrin clot formation is presented with annotated differentially expressed proteins. In green downregulated proteins are shown. These proteins are inhibitors of fibrin clot formation. Thus, fibrin clot formation is activated in diabetes. (http://www.wikipathways.org/index.php/Pathway:WP558).

Hyperglycemia is known to play a critical role in the pathogenesis of cardiovascular disease. Numerous substances, such as growth factors, cytokines and pro-coagulant factors are related to a series of altered underlying processes that induce and promote atherogenesis (Beisswenger, 2012; Katz et al., 2015). In our study, among differentially expressed proteins were PLG, SERPING1, SERPINC1, APOA2, FGB, A2M, which are related to endothelial dysfunction, coagulation processes and pro-atherogenic alteration mechanisms. The differentially expressed proteins involved in the coagulation pathway are illustrated in Figure 4.

Vascular lesions are the result of an unbalance between fibrin deposition and fibrinolysis. Injury in vascular endothelial cells releases plasminogen activators and at the same time activates fibrinolysis. The role of plasminogen activators is to cleave plasminogen into plasmin, which dissolves clots. Fibrinolysis is controlled by plasminogen activator inhibitors (PAI-1) and plasmin inhibitors (a2-macroglobulin) (Beisswenger, 2012). In diabetes, premature atherosclerosis and activation of coagulation factors, combined with hypofibrinolysis all contribute to increased cardiovascular risk. Serin protease inhibitors (SERPINC1, SERPINA1) and A2 macroglobulin are downregulated in poorly control subjects (shown in green) (Figure 4), which further impairs the degradation of fibrin clots (Carr, 2001; Pratte et al., 2009; Chung et al., 2015).

Blood clot formation is the last step in the atherothrombotic mechanism, and the structure of the fibrin network, among other factors, determines cardiovascular risk. Hyperglycemia induces alterations in coagulation factor plasma levels and its impact is crucial in predisposition to cardiovascular events (Katz et al., 2015). Coagulation's deregulation appears to play an important role in glomerular hypertrophy and fibrosis of diabetic nephropathy (Sumi et al., 2011).

In conclusion, by performing analysis at the systems biology level with rigorous statistical methodology this study functional insights by connecting the disease phenotype to specific biological processes (Tables 3, 4). This functional analysis demonstrates the deregulation of biological mechanisms highly relevant to diabetic pathophysiology (inflammation, atherosclerosis signaling, coagulation, etc.) whereas the patients did not exhibit any clinical complications (retinopathy, microalbuminuria, neuropathy) associated with type 1 diabetes. Thus, our study reveals molecular features with clinical relevance that can allow physicians to assess the status of asymptomatic patients.

### Potential preventive intervention

A final step to the bioinformatic analysis was the utilization of L1000CDS^2^ which is a search engine of gene expression signatures from the LINCS L1000 dataset (Vempati et al., 2014) (see Methods). The system is a tool for identifying perturbagens whose overall effect in gene expression either mimics or reverses the gene expression pattern. When provided with the differentially expressed (up & down-regulated) proteins of Comparison G1 vs. G2 and asked to return the agents that most efficiently reversed that phenotype, the top hit was BRD-K01868942 (Supplementary Table 9), a novel serotonin receptor antagonist (Lemaître et al., 2009).

As previously demonstrated on diabetic mice, increased serotonin receptor activity induces contraction of arteries thus causing vascular dysfunction (Nelson et al., 2012). The finding that a serotonin receptor antagonist efficiently reverses our experimental phenotype leads to the suggestion that this phenotype is at least partially induced by increased serotonin receptor activity. The above confirms our aforementioned finding of vascular dysfunction in diabetics with poor glycemic control vs. well-controlled diabetics. Furthermore, this finding suggests that serotonin receptor antagonists could be potentially utilized as a preventive intervention in young patients with poor diabetic control. This possible course of intervention is further supported by the fact that serotonin antagonists improve vascular function in patients with peripheral arterial disease (Miyazaki et al., 2007). Thus, the available pharmacological data on the most prominent predicted active substance support the validity of our bioinformatics approach.

## Conclusion

This study provides the research community with a high quality proteomic resource with state-of-the-art wealth of information for a very specific patient population, which is young individuals with type-1 diabetes and poor glycemic control. In-depth analysis of data from this population indicated that differentially expressed proteins are related to acute phase response, endothelial dysfunction, inflammatory and coagulation processes in type I diabetes mellitus. Furthermore, hyperglycemia appears to be a causal link between diabetes and its complications by activating the respective molecular pathways from the early stages of the disease. Finally, a possible course of preventive intervention was revealed by molecular signatures analysis. The current work enriches the clinical landscape by providing a proof-of-concept on how proteomics and bioinformatics approaches can be applied for the elucidation of molecular pathways involved in the pathophysiology of type 1 diabetes.

## Author contributions

EP collected samples, researched data, ran the proteomic pipeline, performed data analysis, result interpretation and wrote the manuscript. Additionally, EP drew the graphical abstract shown in Figure 1 and edited Figure 4. HV conceived and supervised the study and assisted in manuscript editing. AG-V. assisted with data collection and interpretation. JZ reviewed and significantly edited the manuscript. GM ran the MRM pipeline. KV formulated the study design, supervised experiments, conducted the bioinformatic and statistical analyses and performed results' interpretation. All authors discussed the results, commented on the manuscript and contributed to manuscript preparation and writing.

### Conflict of interest statement

EP was granted an IKY fellowship of excellence for postgraduate studies in Greece- SIEMENS program. The funders had no role in study design, data collection and analysis, decision to publish, or preparation of the manuscript. This does not alter our adherence to Frontiers policies on sharing data and materials. The other authors declare that the research was conducted in the absence of any commercial or financial relationships that could be construed as a potential conflict of interest.
