# Supplementary material for: Saliva Proteomics Analysis Offers Insights on Type 1 Diabetes Pathology in a Pediatric Population
Source: Front Physiol. 2018 Apr 26;9:444. doi: 10.3389/fphys.2018.00444 (PMC5932525; doi:10.3389/fphys.2018.00444)
Supplement: Supplementary file 11 [file Data_Sheet_1.docx]

Supplementary Material

**Saliva Proteomics Analysis Offers Insights on Type 1 Diabetes Pathology in a Pediatric Population**

**Eftychia Pappa^*^, Heleni Vastardis, George Mermelekas, Andriani Vazeou-Gerasimidi, Jerome Zoidakis, Antonia Vlahou, Konstantinos Vougas**

*** Correspondence:** Eftychia Pappa: effiepappa84@yahoo.com

# Supplementary Tables

**Supplementary Table 1.** The total of 36 samples were separated in 6 batches, each batch containing 6 samples two from each group (G1, G2 & Ctrl). Each batch was processed separately as described in the following section and the samples of each batch were iTRAQ-labeled as described in S1 Table.

**Supplementary Table 2**. Spectral information of proteotypic peptides for the design of MRM experiments exist for all proteins and 1-3 peptides per protein were selected

**Supplementary Table 3**. The final transition selection of peptides was based on the quality of the MS/MS spectrum of each peptide in the human spectral library, downloaded from NIST

**Supplementary Table 4**. The proteomic analysis yielded 22028 peptides that were confidently identified (FDR < 1%)( S4 Table) and quantified by the iTRAQ reporter ions.

**Supplementary Table 5**. The identified peptides corresponded to 4876 individual confident protein identifications (FDR< 5%)

**Supplementary Table 6**. The total protein number considered for analysis was reduced to 2031 proteins. Min prob: minimum protein identification probability across all six iTRAQ batches. Max prob: maximum protein identification probability across all six iTRAQ batches.

**Supplementary Table 7**. Functional classification of proteins revealed that Enzymes and Cytokines were the main functional groups of the salivary proteome.

**Supplementary Table 8**. 33 proteins were found to be differentially expressed between G1-Ctrl, 37 between G2-Ctrl and 61 between G1-G2.

**Supplementary Table 9**. We provided L1000CDS^2^, a search engine of gene expression signatures from the LINCS L1000 dataset, with the differentially expressed (up & down-regulated) proteins of Comparison G1 vs G2. Among the agents that most efficiently reversed that phenotype, the top hit was BRD-K01868942, a serotonin receptor antagonist.

**Supplementary Table 10**. Protein Identification in each iTRAQ batch and Protein level false discovery rate estimates by TPP Protein Prophet.
